# Courtship and Mating Behavior of the Aphid Parasitoid *Praon volucre*: Effects of Host Association and Behavioral Lateralization

**DOI:** 10.3390/insects17020192

**Published:** 2026-02-11

**Authors:** Maria C. Boukouvala, Demeter Lorentha S. Gidari, Nickolas G. Kavallieratos

**Affiliations:** Laboratory of Agricultural Zoology and Entomology, Department of Crop Science, Agricultural University of Athens, 75 Iera Odos Str., 11855 Athens, Greece; mbouk@aua.gr (M.C.B.); dlgidari@aua.gr (D.L.S.G.)

**Keywords:** mating sequence, host aphid, parasitic wasp, laterality, Hymenoptera, Braconidae, Aphidiinae

## Abstract

Mating behavior plays a critical role in the reproductive success and population dynamics of insects. *Praon volucre* is a widespread, broadly oligophagous aphid parasitoid and an important natural enemy in agricultural and natural ecosystems. The present study provides the first detailed characterization of the courtship and mating sequence of *P. volucre*, examining the effects of host association and behavioral lateralization on mating performance. Behavioral observations were conducted on virgin *P. volucre* adults emerging from two aphid hosts, *Macrosiphum euphorbiae* feeding on *Citrus aurantium* and *Aulacorthum solani* feeding on *Malva neglecta*. Males from both host-derived populations exhibited a left-side mounting bias, which was associated with faster mate detection, reduced courtship duration, and higher mating success. Mating behavior remained largely stable across host origins, indicating a high degree of behavioral stability. This behavioral robustness, combined with enhanced mating efficiency driven by lateralization, underscores the suitability of *P. volucre* as a biological control agent. The results have practical implications for mass-rearing and release programs, suggesting that a potential host switching during rearing is unlikely to compromise reproductive performance.

## Simple Summary

The present study investigated the influence of lateralization and host association on the mating process and success of two populations of *Praon volucre* emerging from two aphid hosts, *Macrosiphum euphorbiae* and *Aulacorthum solani*. A left-biased mounting tendency, associated with a faster mating process and higher copulation success, was observed for both host-derived populations. The investigation of the host association did not lead to significant differences between the two *P. volucre* populations. These findings indicate that *P. volucre* has a stable and efficient mating system. Understanding mating behavior can therefore help improve mass-rearing strategies and the effectiveness of parasitoid-based aphid control.

## 1. Introduction

Insect population dynamics, reproductive success, and evolutionary paths are all significantly influenced by mating behavior [1]. Since adults of parasitic Hymenoptera usually have short lifespans, successful mating is very important [2,3,4,5,6]. A series of visual, tactile, and auditory cues, such as wing fanning, antennal tapping, and mounting, frequently appear in parasitoid courtship behaviors to help mate detection and copulation [3,7,8,9].

Aphidiinae parasitoids (Hymenoptera: Braconidae) are among the most important natural enemies of aphids in agricultural and natural ecosystems, playing a major role in the biological control of aphid infestations [10]. *Praon volucre* (Haliday) (Hymenoptera: Braconidae: Aphidiinae) is the most frequent *Praon* species in European cereal agroecosystems [10]. It is a broadly oligophagous species that attacks a variety of aphid hosts [11]. While extensive research has focused on its taxonomy, biodiversity, host associations, and foraging behavior [11,12,13,14,15], there is an absence of information on the mating behavior of this species.

Aphids are herbivorous hemipterans belonging to the family Aphididae (Aphidoidea). Many of them cause economic damage, directly by sap consumption or indirectly by transferring plant viruses [16]. Less than 1% of aphid species are able to feed on plant species belonging to multiple taxonomic families; the majority are specialized in host plants that belong to closely related taxonomic groups within the same family [17,18]. Aphid species frequently exhibit genetic differences among their populations based on host plants, leading to the existence of several biotypes that are specialized to feed on a small number of host plants [16]. Control of aphids is difficult as they can quickly penetrate and damage plants due to their large-scale reproduction [19,20]. *Macrosiphum euphorbiae* (Thomas) has a broad host range of more than 200 plant species belonging to 20 different families, including ornamental and herbaceous crops [21]. Plant viruses, including potato leafroll virus (PLRV) and potato virus Y (PVY), are also transmitted by *M. euphorbiae* [22]. Native to Europe, *Aulacorthum solani* (Kaltenbach) is an economic pest currently widespread in America, Asia, Africa, and Oceania [23,24,25,26,27]. It is a polyphagous aphid with up to 95 host species belonging to 25 families [28].

Several parasitoid characteristics, including behavioral traits, may be affected by the host aphid species. Host-associated variation has been documented in several morphological and biological traits of aphid parasitoids, such as body size, developmental rate, and fitness-related parameters [29,30,31,32]. Behavioral characteristics, like mating behavior or courtship, may also be affected by such host-induced effects [7]. In addition, behavioral lateralization is an important aspect of animal behavior, including insects [33]. Lateralized behaviors (e.g., escape and surveillance asymmetries, visual learning, left or right bias of exploring or kicking, direction of mounting during copulation), which emerge as an individuals’ preference to use one side of the brain (left or right) during their activities, provide various advantages related to the organization of their brains and evolution [33,34,35,36,37,38]. In some insect taxa, lateralization has been linked to enhanced copulation success during mating [8,39,40,41].

Given the importance of *P. volucre* in aphids’ biological control and its understudied behavior, the present study aims to provide the first detailed description of the courtship and mating behavior of this species. Thus, the objective of the study was to quantify the observed mating traits and examine whether they are influenced by the lateralization and the different aphid hosts.

## 2. Materials and Methods

### 2.1. Collection and Rearing of Insects

Fifty colonies of *M. euphorbiae* feeding on *Citrus aurantium* L. (Rutaceae) or *A. solani* feeding on *Malva neglecta* Wallr. (Malvaceae) were collected alive and mummified in Athens during April and May. In order to identify aphids using the keys of Blackman and Eastop [42,43], the collected material was placed in plastic tubes wrapped with thin elastic gauze and transferred to the Agricultural University of Athens, Greece (Laboratory of Agricultural Zoology and Entomology). The only aphids found were *M. euphorbiae* and *A. solani*. Aphids were kept in a 90/75% ethanol/lactic acid solution [44]. After being tightly sealed, the plastic containers were moved into boxes with openings so that their contents could be adequately aerated. The plant-aphid-mummy material was kept at 22 °C, 55% relative humidity, and a 16:8 (L:D) photoperiod. Using a gentle brush, each mummy was meticulously placed into gelatin capsules along with a small plant-delivered part. On a daily basis, the capsules were examined for the emergence of parasitoids. Using an Olympus stereomicroscope (SZX9) (Olympus, Tokyo, Japan) and the taxonomic keys of Ghaliow et al. [13] and Kavallieratos et al. [45], adult parasitoids were identified at the species level. One species, *P. volucre*, was identified parasitizing *M. euphorbiae* and *A. solani*. For the emerging parasitoids to reach sexual maturity, they were kept for 2 days in Petri dishes (diameter of 5 cm, height of 1 cm) with honey, pollen, and unlimited water on filter paper with a diameter of 1 cm [7,46].

### 2.2. Behavioral Observations

Behavioral observations were carried out in a room with daylight fluorescent tubes (approx. 22 °C; 55% RH). Trials took place in an arena (ø = 60 mm) between 10:00 and 16:30. The parasitoids under observation were between two and four days old [46]. For each replication, new parasitoids of the same age were used. To assess the courting and mating behavior of *P. volucre*, two glass vials were used to safely transfer a virgin female and a virgin male to the arena. Male behavior was observed for 20 min (or until mating was finished) using an Olympus (SZX9) (Olympus, Tokyo, Japan) stereomicroscope. For each replication, the duration of the subsequent phases was recorded: (i) mate detection (time the male needed to detect his mate), (ii) wing fanning (time where the male fluttering his wings towards the female) [8], (iii) chasing (time spent by the male following the female), (iv) pre-copulation time (time spent by the male trying to mount the female until the contact of genitalia), (v) antennal tapping (time where the male palpating, using his antennae, the body of the female), (vi) copula (from the insertion of the male’s aedeagus into the female’s genital chamber until separation). Additionally, behavioral asymmetries at the population level were also investigated, examining the mounting side. Whether this behavioral asymmetry influenced courtship and mating traits was also investigated. Both successful and unsuccessful mating attempts were noted. Pairs that did not perform any courtship or did not move for longer than half an hour were discarded. One pair (one virgin male and one virgin female) was observed each time, and by the end of the copulation, each pair was discarded. A total of 44 pairs of *P. volucre* parasitizing *M. euphorbiae* and 47 pairs of *P. volucre* parasitizing *A. solani* were used for the analysis.

### 2.3. Statistical Analysis

The laterality behavior of *P. volucre* was analyzed using the JMP 16.2 software [47]. The Steel-Dwass test (significance level of α = 0.05) was used to examine the effects of mounting lateralization on the duration of the mating traits of *P. volucre* emerging from *M. euphorbiae* or *A. solani*, which did not follow a normal distribution. The same test was used to determine the mean duration of each behavioral mating trait of *P. volucre* emerging from *M. euphorbiae* or *A. solani* regardless of lateralization. The JMP 16.2 software [47] was used to analyze data on *P. volucre* mating success using a weighted generalized linear model with binomial distribution: *y* = *Xβ* + *ε*. *y* is the observations’ vector (i.e., successful or unsuccessful copulation), *X* is the incidence matrix, *β* is the fixed effect’s vector (i.e., the mounting side), and *ε* is the random residual effect’s vector. The significance of value differences was assessed at a probability level of 0.05.

## 3. Results

### 3.1. Courtship and Mating Sequence of P. volucre Parasitizing M. euphorbiae

**Figure 1:**
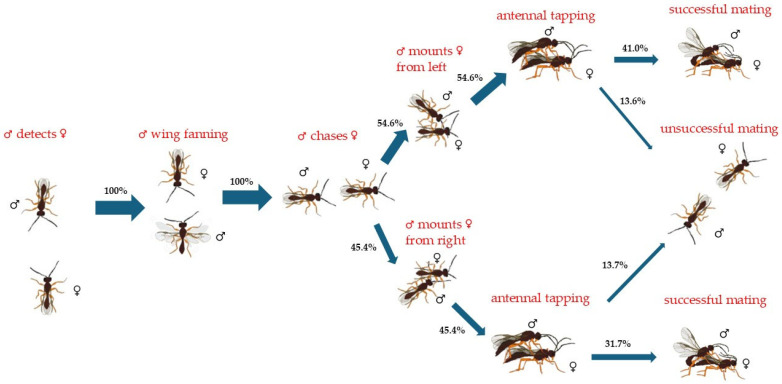
Flow chart of mating and courtship behavior of *Praon volucre* male adults emerging from *Macrosiphum euphorbiae*. The percentage of insects exhibiting each behavior is demonstrated by the width of each arrow (*n* = 44 pairs).

Most of the *P. volucre* parasitizing *M. euphorbiae* (54.6 out of 100%) mounted the females from their left side and the rest of them (45.4 out of 100%) from the right side. The left-biased males achieved 41.0 out of 54.6% successful copulations, while the right-biased males achieved 31.7 out of 45.4%. The unsuccessful copulation rates for the left- and right-biased males were 13.6 out of 54.6% and 13.7 out of 45.4%, respectively (Figure 1).

**Figure 2:**
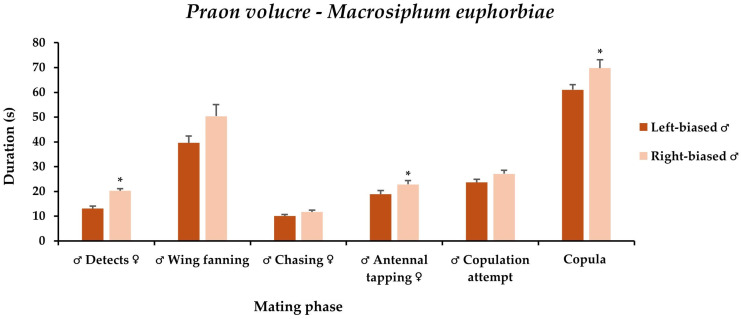
**The** mean time for each mating trait of *Praon volucre* male adults emerged from *Macrosiphum euphorbiae*, characterized by the side-bias of mounting. Within each trait (for ♂ Detects ♀ *χ*^2^ = 25.5; df = 1; *p* < 0.001; for ♂ Wing fanning ♀ *χ*^2^ = 2.5; df = 1; *p* = 0.12; for ♂ Chasing ♀ *χ*^2^ = 2.7; df = 1; *p* = 0.10; for ♂ Antennal tapping ♀ *χ*^2^ = 4.5; df = 1; *p* = 0.03; for ♂ Copulation attempt *χ*^2^ = 3.4; df = 1; *p* = 0.07; for Copula *χ*^2^ = 4.0; df = 1; *p* = 0.05), significant differences are indicated by the asterisks (Steel-Dwass test, *p* < 0.05).

The laterality of mounting significantly impacted mate detection time, antennal tapping duration, and copulation time. Left-biased males detected their mates significantly faster (13.1 s) than the right-biased (20.3 s). Concerning the antennal tapping time, the left-biased males performed this trait for significantly shorter time (18.8 s), compared to those that mounted from the right side of the females (22.8 s). The left-biased *P. volucre* males copulated for a significantly shorter time (61.1 s) than the right-biased (69.8 s) (Figure 2).

### 3.2. Courtship and Mating Sequence of P. volucre Parasitizing A. solani

**Figure 3:**
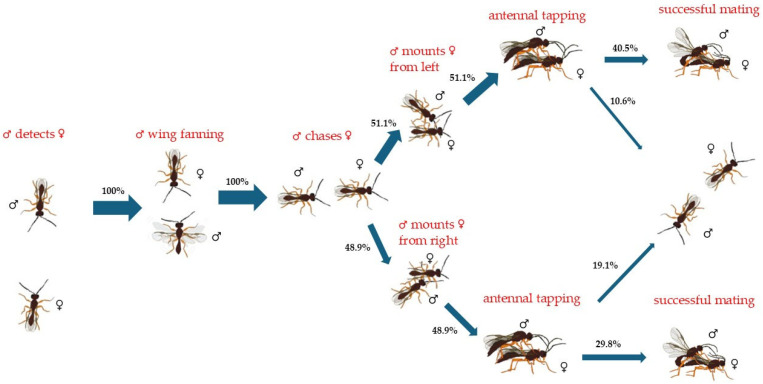
Flow chart of mating and courtship behavior of *Praon volucre* male adults emerged from *Aulacorthum solani*. The percentage of insects exhibiting each behavior is demonstrated by the width of each arrow (*n* = 47 pairs).

Similar to the *M. euphorbiae*-parasitizing *P. volucre* males, most of the *P. volucre* males parasitizing *A. solani* (51.1 out of 100%) mounted their mates from their left side, achieving 40.5 out of 51.1% successful copulations and failing at 10.6 out of 51.1%. Concerning the right-biased males (48.9 out of 100%), they resulted in 29.8 and 19.1 out of 48.9% successful and unsuccessful copulation rates, respectively (Figure 3).

**Figure 4:**
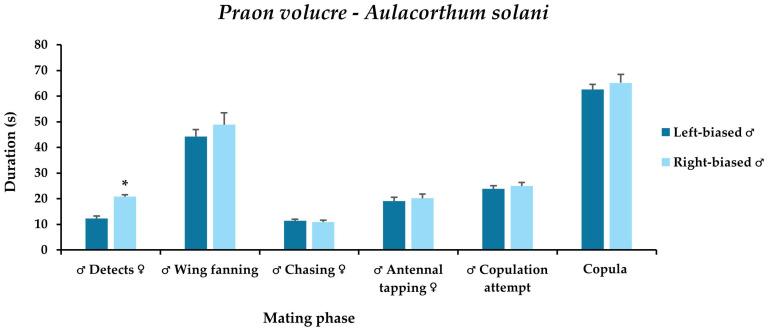
**The** mean time for each mating trait of *Praon volucre* male adults emerged from *Macrosiphum euphorbiae*, characterized by the side-bias of mounting. Within each trait (for ♂ Detects ♀ *χ*^2^ = 29.8; df = 1; *p* < 0.001; for ♂ Wing fanning ♀ *χ*^2^ = 0.7; df = 1; *p* = 0.40; for ♂ Chasing ♀ *χ*^2^ = 0.4; df = 1; *p* = 0.53; for ♂ Antennal tapping ♀ *χ*^2^ = 0.1; df = 1; *p* = 0.83; for ♂ Copulation attempt *χ*^2^ = 0.1; df = 1; *p* = 0.73; for Copula χ^2^ = 0.7; df = 1; *p* = 0.41), significant differences are indicated by the asterisks (Steel-Dwass test, *p* < 0.05).

The laterality of mounting significantly affected only the mate detection time. Left-biased males detected their mates significantly faster (12.3 s) than the right-biased (20.8 s). The other mating traits (wing fanning, chasing, antennal tapping, copulation attempts, and copula) did not significantly differ, although left-biased males were faster than the right-biased at all traits except for the chasing time (Figure 4).

### 3.3. Praon volucre Emerging from M. euphorbiae vs. P. volucre Emerging from A. solani

**Figure 5:**
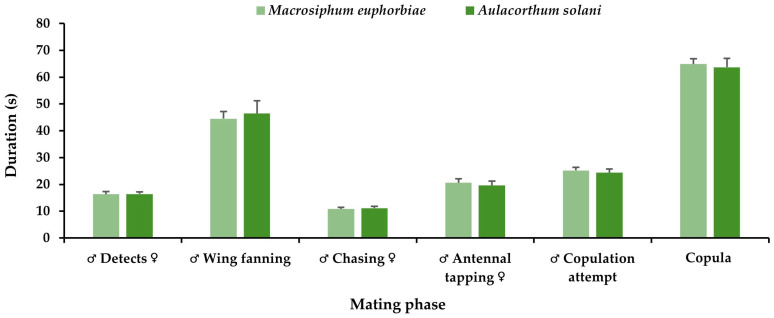
Mean time for each mating trait (for ♂ Detects ♀ *χ*^2^ = 0.002; df = 1; *p* = 0.97; for ♂ Wing fanning ♀ *χ*^2^ = 0.4; df = 1; *p* = 0.54; for ♂ Chasing ♀ *χ*^2^ = 0.1; df = 1; *p* = 0.71; for ♂ Antennal tapping ♀ *χ*^2^ = 0.5; df = 1; *p* = 0.46; for ♂ Copulation attempt *χ*^2^ = 0.3; df = 1; *p* = 0.57; for Copula *χ*^2^ = 0.04; df = 1; *p* = 0.84) of *Praon volucre* male adults emerged from *Macrosiphum euphorbiae* or *Aulacorthum solani* (Steel-Dwass test, *p* < 0.05).

Comparing the time-related mating traits of *P. volucre* emerging from *M. euphorbiae* and from *A. solani*, there were no significant differences between the two tested populations. However, *P. volucre* males emerging from *M. euphorbiae* detected their mates faster (16.3 vs. 16.4 s), performed wing fanning (44.5 vs. 46.5 s) and chased (10.8 vs. 11.1 s) the females for less time, compared to *P. volucre* males emerging from *A. solani*, while the opposite was observed for the antennal tapping (20.6 vs. 19.6 s), the copulation attempts time (25.2 vs. 24.4 s), and the copula duration (64.9 vs. 63.7 s) (Figure 5).

**Figure 6:**
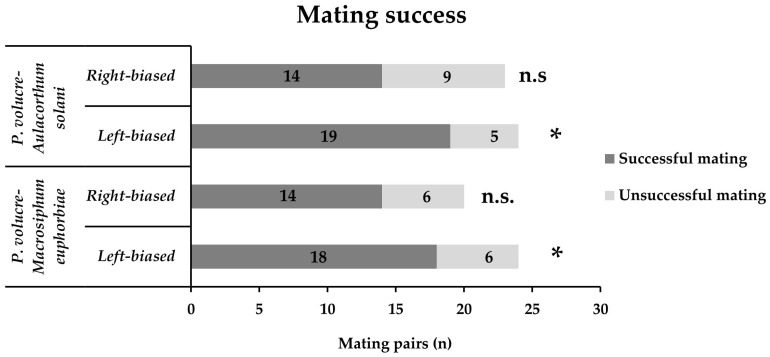
**The** copulation success of *Praon volucre* male adults emerged from *Macrosiphum euphorbiae* or *Aulacorthum solani*. Significant differences (generalized linear model, binomial distribution, *p* < 0.01) are indicated by asterisks; n.s. stands for not significant.

The mating success of both populations is presented in Figure 6. Mounting laterality did not significantly affect the male mating success (*χ*^2^ = 0.137, df = 1, *p* = 0.71, and *χ*^2^ = 1.898, df = 1, *p* = 0.17 for *P. volucre* male adults emerged from *M. euphorbiae* and *A. solani*, respectively). However, males of both populations that performed left-biased mountings showed higher copulation success (*χ*^2^ = 6.042, df = 1, *p* < 0.01, and *χ*^2^ = 8.208, df = 1, *p* < 0.01 for *P. volucre* male adults emerged from *M. euphorbiae* and *A. solani*, respectively), while right-biased mountings of males of both populations resulted in lower copulation success (*χ*^2^ = 3.250, df = 1, *p* > 0.05, and *χ*^2^ = 1.130, df = 1, *p* > 0.05 for *P. volucre* male adults emerged from *M. euphorbiae* and *A. solani*, respectively).

## 4. Discussion

The results of the present study demonstrated that both behavioral lateralization and host origin influence key components of mating performance. Both parasitoid populations took advantage of the left-biased mounting, reducing the whole mating sequence time and achieving higher rates of successful copulation. However, differences observed between the two populations indicate a host-associated influence on the mating performance.

The mating sequence (mate detection followed by wing fanning, chasing, precopulatory mounting, antennal tapping, and copulation), observed in both *P. volucre* populations, follows the pattern of other Braconidae species, belonging to genera *Cardiochiles*, *Aphidius*, *Cotesia*, *Glyptapanteles*, and *Diaeretiella* [46,48,49,50,51,52,53,54,55]. Wing fanning and antennal tapping appear to play a crucial role in sexual communication, likely facilitating female receptivity through visual, mechanical, and possibly chemical cues [7,49,51,52,55,56,57]. Given the relatively short lifespan of parasitoid hymenopteran parasitoids, including *P. volucre* [4,5,6], such cues that facilitate and accelerate copulation allow males to maximize mating opportunities. Based on the results of the present study, faster wing fanning and antennal tapping performances lead to faster and more successful copulation rates. Whether this is a general trait in aphidiines needs further investigation.

A key finding of this study is the consistent advantage of the left-biased males in both host-derived populations. Left-biased males detected females significantly faster and performed courtship and copulation phases in less time than the right-biased males. Additionally, left-biased mounting positively influenced the mating success rates at both populations. These results support the growing body of evidence indicating that lateralization enhances motor coordination and behavioral efficiency in insects [38,39,40,41,58,59,60]. In *P. volucre*, left-biased males may process female cues more efficiently, resulting in faster and more successful copulations. The presence of this bias in both host-derived populations indicates that lateralization is likely a species-level trait rather than a host-induced trait.

Although no statistically significant differences were detected in the duration of mating traits between *P. volucre* males emerging from *M. euphorbiae* and *A. solani*, consistent trends were evident. Males emerging from *M. euphorbiae* tended to detect females faster, performed wing fanning, and chased the females for less time, whereas males from *A. solani* performed shorter antennal tapping, copulation attempts, and copula durations. Additionally, their common left bias influenced their mating sequence differently, resulting in a more vigorous effect for the *P. volucre* males emerging from *M. euphorbiae*. These patterns suggest subtle host-associated effects on behavioral expression, potentially mediated by differences in host quality, nutritional content, or developmental conditions. Host-associated variation is well documented in aphid parasitoids for traits such as body size, development time, and fitness [29,30,31,32]. Considering the aforementioned host-associated results of the present study, mating behavior may be more stable and therefore less sensitive to host origin. This behavioral stability may be adaptive for a widely oligophagous species like *P. volucre* [11], enabling effective reproduction across a broad range of aphid hosts and host plants.

This behavioral consistency is particularly advantageous for biological control programs, where parasitoids may be reared on one aphid species but released into crops infested by different aphids. The lack of significant host-induced influence in mating behavior indicates that host switching during mass rearing is unlikely to compromise mating performance after release. Selecting rearing hosts that promote faster mate detection and reduced courtship time could enhance mating rates and population growth in mass-produced parasitoids, given the strong link between the mating behavior and the reproductive output [61,62]. In this context, it is very important to take advantage of a broad-range parasite, such as *P. volucre*, and to study the mating sequence of this species in more aphid hosts. Between the two tested hosts of the present study (*M. euphorbiae* and *A. solani*), the whole mating sequence duration slightly differed, with *P. volucre* males emerging from *A. solani* performing faster mating. Detecting a host where *P. volucre* can achieve copulation in notably shorter time could lead to higher population dynamics in mass rearing production. Additionally, since long-term mass rearing in a single host negatively influences insects’ courtship [63,64,65], the ability to change the host in order to maintain short time and high rates of successful copulation can help counteract the negative effects of mass production. Thus, *P. volucre,* which has an extensive host range [11], is an advantageous case of biological control.

## 5. Conclusions

This study demonstrates that *P. volucre* has a distinct left-side mating bias and a structured courtship sequence that improves mating success and efficiency. These results emphasize the significance of lateralization as a factor determining mating performance and advance the comprehension of the behavioral ecology of aphidiine parasitoids. Future studies are required to link the mating behavior to fecundity, offspring sex ratio, and field performance, further clarifying the ecological and applied significance of behavioral lateralization in biological control agents. Furthermore, it would be interesting to study the molecular mechanism of the lateralization, related to *P. volucre*, and the effect of cross-mating on a parasitoid species derived from two different hosts.

## Data Availability

The original contributions presented in this study are included in the article. Further inquiries can be directed to the corresponding author.
